# Levels and predictors of participation in integrated treatment programs for pregnant and parenting women with problematic substance use

**DOI:** 10.1186/s12889-019-6455-4

**Published:** 2019-02-06

**Authors:** Thao Lan Le, Chris Kenaszchuk, Karen Milligan, Karen Urbanoski

**Affiliations:** 1 Institute for Mental Health Policy Research, Centre for Addiction and Mental Health, 33 Russell Street, Toronto, ON M5S 2S1 Canada; 2 Department of Psychology, Ryerson University, 350 Victoria Street, Toronto, ON M5B 2K3 Canada; 3 Canadian Institute for Substance Use Research, Public Health and Social Policy, University of Victoria, 2300 McKenzie Avenue, Victoria, BC V8W 2Y2 Canada

**Keywords:** Treatment participation, Treatment retention, Integrated programs

## Abstract

**Background:**

Women who are seeking services for problematic substance use are often also balancing responsibilities of motherhood. Integrated treatment programs were developed to address the diverse needs of women, by offering a holistic and comprehensive mix of services that are trauma- and violence-informed, and focus on maternal and child health promotion and the development of healthy relationships.

**Methods:**

Using system-level administrative data from a suite of outpatient integrated programs in Ontario, Canada, we described the clients and rates and predictors of treatment participation over a 7-year period (2008–2014; *N* = 5162).

**Results:**

All participants were either pregnant or parenting children under 6 years old at admission to treatment. Retention (length of time between the first and last visit) averaged 124.9 days (SD = 185.6), with episodes consisting of 14.6 visits (SD = 28.6). The vast majority of women attended more than one visit (87.2%), typically returning within 2 weeks (mean 12.3 days, SD = 11.1). In addition to being pregnant or new mothers experiencing problematic substance use, most were unemployed, on social assistance, and single.

**Conclusions:**

Programs appeared to be able to successfully engage most women in treatment once they accessed the programs. Although rates of treatment participation did vary across subgroups defined by sociodemographic and admission characteristics, effect sizes tended to be small on average, providing little evidence in general of sociodemographic inequities in participation. Further work is needed to study the influence of program-level factors on participation, and how these link to maternal and child outcomes.

## Background

Women who are seeking services for problematic substance use are often also balancing responsibilities of motherhood. Integrated treatment programs were developed to address the diverse needs of women, by offering a variety of services either on-site or through partnering agencies. To the extent that these programs are able to better meet the needs of women and their children, they can be expected to be more effective in engaging women in treatment, relative to traditional substance use services [1]. That said, studies of participation in integrated programs are sparse, and little is known about how levels of participation vary across subgroups of the population. This population-based study investigates participation in integrated programs within a system of care in Ontario, Canada. By examining key indicators of treatment attendance and retention, this study contributes to the literature on integrated programs and helps to pinpoint potential inequities in women’s ability to engage with services.

### Integrated treatment for women who are pregnant and parenting

Integrated programs for pregnant and parenting women attempt to reduce common barriers that make it difficult for women to participate in substance use services (e.g., lack of childcare, fear of loss of child custody, limited services for pregnant women, provider stigma) [2–6]. They are designed to offer a holistic and comprehensive mix of services that are trauma- and violence-informed, and focus on maternal and child health promotion and the development of healthy relationships [7–10]. Programs seek to overcome the traditional fragmentation of service sectors, offering counselling for substance use and mental health, prenatal and primary care, services for parenting and child development, and other supports to address social-economic resources (e.g., food security, housing). Meta-analyses have shown that integrated programs are equivalent to standard care in terms of reduced maternal substance use, and superior in terms of improved maternal mental health, attendance at prenatal visits, parenting, and birth and child outcomes [1, 9, 11–13]. As such, integrated treatment programs play a key role in the care continuum, with important public health implications related to women’s health and well-being, as well as fetal and child development [7, 14].

### Participation in integrated programs

Across a variety of substance use treatment modalities (e.g., opioid substitution therapy, outpatient counselling, and residential programs), longer retention or length of stay typically predicts better post-treatment outcomes, defined by reduced substance use [15–17]. At an individual-level, retention is driven by a host of personal and program-level factors and the interactions between them (e.g., the extent to which a given program fits with a person’s needs, strengths, and expectations). It is in this sense that measures of treatment attendance can be seen as important indicators of treatment process and service quality, and developing strategies to prolong retention have become a priority for system planners and service providers [18–21]. In outpatient settings where gaps between visits can complicate the interpretation of overall length of affiliation with a program, measures that incorporate the timing and intensity of visits are required to accurately characterize levels of participation. In outpatient settings, shorter intervals between appointments early on in the treatment episode have been shown to promote better engagement in services [22, 23].

Studying variation in treatment participation across subgroups of the treatment population enables identification of potential gaps in the system, providing data to inform program development and quality improvement efforts [24, 25]. There is evidence that factors such as younger age, lower socio-economic status, greater substance use, and injection drug use are associated with premature dropout and shorter retention in treatment generally [26–30]. Others have shown that, among women more specifically, higher income, being married, and being unemployed predict longer retention [31]. Being younger, single, and having low socioeconomic status may all affect women’s abilities to access and participate in programs, particularly if childcare and transportation needs are not met. Structural and stigma-related barriers to care are also compounded when coupled with socioeconomic disadvantage [32, 33], which can be expected to further affect women’s access to services.

There is a gap in research assessing levels of participation in integrated treatment for pregnant and parenting women, and no studies have looked at predictors of participation within systems of care. In their meta-analysis, Milligan et al. [1] found longer retention in integrated programs than in standard programs, although rates of treatment completion did not differ. There is a need for work that characterizes patterns of participation in integrated programs and investigates variation across key population subgroups, as part of broader efforts to evaluate equity in engagement in services within the population.

### Context and objectives

In 2002, the government of Ontario, Canada funded an initiative through the Ontario Early Years Strategy to increase the capacity of existing substance use treatment agencies to meet the needs of pregnant and parenting women. Agencies developed their own services designed to strengthen ties between substance use and related health and social services, tailored to community needs and resources. The resulting programs share a target population of women who are pregnant or parenting children under 6 years old and who identify as having substance-related problems, although they vary in terms of specific interventions, staffing, and service models. In addition to counseling for substance use, these programs variously include mental health counseling, prenatal and primary care, parenting support, childcare, case coordination with child protective and legal services, life skills training, and supports for food, employment, and housing [34]. All are offered on an outpatient basis, with scheduled appointments and groups. Programs do not have a designated length (i.e., services are offered for as long as women wish to stay engaged). Stakeholders (including researchers, service providers, administrators, and health planners who work in the area of women’s health and substance use) described the care in these programs as women-centred, empowering, holistic, and focused on meeting the needs of women and their children [35]. Programs work toward helping women to reduce their substance use or maintain abstinence, with other intended outcomes relating to parenting and child custody, housing, social supports, and improved maternal and child health [36, 37]. As of 2014, there were 36 such programs operating throughout Ontario.

Despite being available for more than a decade, little evaluation work has been conducted with this suite of programs. Embedded within a larger system of care, they provide a valuable opportunity to evaluate capacity to engage pregnant and parenting women in services for substance use and to examine equity in participation. The objectives of this study were to: 1) describe the population of women attending integrated programs in Ontario; and 2) evaluate levels and predictors of treatment participation. This study forms part of a larger mixed methods evaluation of treatment processes and outcomes in Ontario’s integrated programs [35, 38].

## Methods

### Data

Data for this secondary analysis were extracted from the Drug and Alcohol Treatment Information System (DATIS; www.datis.ca). DATIS is a centralized information system that collects data on client characteristics and service use from publicly funded addiction treatment agencies in Ontario [39]. Participating agencies (approximately 170) provide a mix of outpatient, residential, and withdrawal management services, which are covered through the province’s universal health insurance program and accessed free of charge by residents. DATIS does not collect information from services provided in hospitals, private treatment clinics, by physicians (e.g., in primary care settings), or by mental health care providers. Self-help groups (e.g., Alcoholics Anonymous/Narcotics Anonymous) are also excluded.

DATIS utilizes a web-based platform for data entry and management. Service providers enter data at admission after the initial appointment, including sociodemographic information, substance use, and other admission details. The probabilistic matching algorithm within Oracle (UTL_MATCH) was used to identify and link records across clients in the database, based on provincial health card number, gender, first and last name, last name at birth, and date of birth [40]. DATIS staff conduct systematic data quality checks and collaborate with the service providers to review data annually.

We extracted de-identified data from the 36 agencies that offered integrated programs. A number of agencies with integrated programs were excluded because they did not use the DATIS clinical-tracking module to record outpatient visit dates, needed to quantify treatment participation (*n* = 7 of 36). Study data from 29 agencies cover the seven-year period from April 1, 2008 to March 31, 2015 (*N* = 7352 treatment episodes for 5162 women). All participants are women who were pregnant or parenting children under 6 years old at admission. A treatment episode was defined as a series of one or more visits separated by intervals of less than 60 days, i.e., a 60-day service-free period indicated the start of a new episode [18, 41]. We selected this seven-year study period for the broader evaluation of treatment processes and outcomes, as it corresponds to a period of stable funding for this suite of programs. In addition, by 2008, agencies had transitioned to using a web-based data entry and management for DATIS.

The institutional Research Ethics Boards of the Centre for Addiction and Mental Health and Ryerson University approved the study.

### Measures

We calculated three episode-level measures of treatment participation: *first visit interval* (number of days between the first and second visit), *retention* (number of days between the first and last visit), and *intensity* (number of visits per episode). By definition, episodes that consisted of only one visit are excluded from the calculation of *first visit interval*. Single-visit episodes were assigned values of 0 for *retention* and 1 for *intensity*.

Client-level measures included age (continuous), education (less than high school vs. high school or more), employment status (employed full- or part-time vs. unemployed or not in the labour force), social assistance (received provincial income or disability support vs. no), marital status (single, widowed, divorced, or separated vs. married or partnered), and pregnancy status (yes vs. no or unsure, where unsure includes women who reported possibly or unknown). Involvement with the legal system was captured as a dichotomous variable (yes vs. no, where yes referred to awaiting trial or sentencing or being on probation or parole at the time of admission). Treatment mandates were based on whether a third party required the client to enter treatment, coded as: no mandate, legal mandate (a choice between treatment or jail, or treatment as a condition of probation or parole), child protection mandate, or mandate from an employer or school.

For each treatment episode, clients can identify up to 5 problem substances (i.e., substances that they want to address during treatment) from a list of 17 substance types. For each substance that they endorse as a problem, they are then asked to indicate how often they used it in the past month. We created dichotomous indicators to denote problems with alcohol, stimulants (powder cocaine, crack, other amphetamines including methamphetamine), cannabis, and opioids (prescription opioids, heroin, opium, and over-the-counter-codeine), which were the top 4 problem substances that women reported. Past-month frequency of use of problem substances was coded as: none, up to twice weekly, three or more times per week, and binge use (referring to periodic excessive use). For those who reported multiple problem substances, the highest frequency reported was used. Past-year non-medical injection drug use was coded as yes vs. no. All variables were self-reported by clients.

### Analysis

We described the sociodemographic, substance use, and admission characteristics of women admitted to integrated programs, and calculated summary statistics for first visit interval, retention, and intensity. We used multilevel regression to model the predictors of the three participation variables, all of which are positively skewed count variables with no negative values. In a situation of positive skewness and variance much larger than the mean, using a statistical model that assumes the presence of normally distributed residuals will often be inappropriate because it can lead to incorrect confidence intervals and *p* values [42]. Instead, regression models for count data are usually more appropriate, e.g., Poisson and negative binomial models. We examined the unconditional means and variances of the participation variables and decided that count regression models could be suitable. To determine the optimal model form, we calculated model-predicted probabilities for outcome values of 1–10 (0–10 for retention) from Poisson and negative binomial regressions, including zero-inflated versions [43], and plotted them against observed probabilities. For all 3 outcomes, predicted probabilities from the negative binomial models best resembled observed probabilities.

PROC GLIMMIX in SAS v.9.3 was used to run multilevel negative binomial regression models, accounting for the clustering of clients within treatment agencies. The first treatment episode per person was selected for analysis. Predictor variables were entered as fixed effects, with a random effect for agency. Variables with statistically non-significant coefficients were removed from the models (alpha = 0.05). Changes in model fit after deleting fixed effects were examined using the − 2 loglikelihood (−2LL), Akaike information criterion (AIC), and Schwarz Bayesian information criterion (BIC), for which lower values indicate better fit.

Missing values ranged from 0 to 4.2% among the predictor variables (Table 1). Records with missing values were excluded from the regression models (final n for each model shown in the tables).

**Table 1:** Characteristics of women attending integrated programs in Ontario (2008–09 to 2014–15)^a^

Characteristics	N	%
Education:		
Less than high school	2332	45.2
High school or more	2677	51.9
Missing	153	2.9
Receiving social assistance:		
No	1645	31.9
Yes	3296	63.9
Missing	221	4.2
Employment status:		
Not employed	4331	83.9
Employed full or part time	732	14.2
Missing	99	1.9
Marital status:		
Married or partnered	1622	31.4
Not married	3491	67.6
Missing	50	1.0
Pregnant at admission:		
No, possibly, or unknown	4168	80.7
Yes	974	18.9
Missing	20	0.4
Problem substance(s): ^b^		
Alcohol	2259	43.8
Stimulants ^c^	2124	41.2
Cannabis	1980	38.4
Opioids ^d^	1617	31.3
Past-year injection drug use:		
No	4591	88.9
Yes	551	10.7
Missing	20	0.4
Maximum frequency of substance use, past 30 days:		
Binge use	148	2.9
3 times per week to daily	2164	50.6
Up to 2 times per week	857	16.6
No use	1466	28.4
Missing	77	1.5
Legal system involvement:		
No	3798	73.6
Yes	1247	24.2
Missing	117	2.2
Treatment mandate:		
None	3233	62.6
Legal system	221	4.3
Child protection services	1444	28.0
Employer or school	143	2.8
Missing	121	2.3

^a^First admission to treatment during the study period; all characteristics self-reported by clients at admission. N = 5162 clients

^b^Clients selected problem substances; categories are not mutually exclusive (percentages do not sum to 100%)

^c^Includes powder cocaine, crack, other amphetamines including methamphetamine

^d^Includes prescription opioids, heroin, opium, and over-the-counter codeine

## Results

### Characteristics of clients at admission

The mean age of women entering integrated programs was 28.7 years (SD = 7.5, min-max = 13–78). Post-hoc analysis revealed that 28 women (< 1%) were older than 55 at admission. Given that the programs are intended to be eligible for women who are pregnant or parenting children under 6 years old, this means that women older than 55 would have given birth at age 50 or older. Although this is possible, these values may also represent measurement or data entry errors, relatives with parenting responsibilities, or women who were otherwise deemed likely to derive benefit from participation in the program. Just over half of women had a high school diploma, two-thirds were receiving social assistance, and 83.9% were not employed (Table 1). Most were not married or partnered, and 18.9% reported being pregnant at admission. Roughly 40% reported problems with (each of) alcohol, stimulants, and cannabis, and one-third reported problems with opioids, although a sizable minority (28.4%) reported no recent substance use at admission. Substance use between 3 times per week and daily was the most commonly reported frequency in the month prior to admission (reported by 50.6%). Ten percent reported past-year injection drug use. One in four women were involved in the legal system and approximately one-third were mandated to attend treatment, most commonly from child protection services. Mandates from the legal system and employers or school authorities were rare.

### Treatment participation

A small proportion of women (14.1%) did not return for a second visit. Among those who did return, 50% were seen a second time within 8 days (mean = 12.3 days; Table 2). Mean episode length (retention) was 124.9 days, during which time clients were seen an average of 14.6 times (intensity). This averages to one visit per 8.4 days, or roughly one visit per week.

**Table 2:** Characteristics of episodes of integrated programs in Ontario (2008–09 to 2014–15) ^a^

Treatment participation	Mean	Std. Dev.	Median
First visit interval: ^b^ Number of days between first and second visit	12.3	11.1	8
Retention: Number of days between first and last visit	124.9	185.6	58
Intensity: Number of visits per episode	14.6	27.7	6

^a^Episodes defined as a series of 1 or more visits separated by intervals of < 60 days. N = 7352 episodes

^b^Excludes episodes consisting of only 1 visit (*N* = 1036, 14.1% of 7352)

Older age was associated with a shorter interval between the first and second visits (Table 3). Regression-predicted values are reported for 2 ages to aid interpretation: women aged 25 were predicted to have a second visit in 12.5 days, relative to 10.8 days among women aged 40. The interval between the first and second visit was 1 day longer for women who were on social assistance or reported problems with alcohol or opioids (relative to the reference categories for these variables). Past-month binge use or (near) daily substance use and past-year injection drug use were associated with 2 fewer days between the first and second visits (relative to women with no past-month use or no injection). The interval was also 3 days *longer* for women mandated by the legal system, and 1 day longer for women mandated by child protection (relative to those with no mandates).

**Table 3:** Predictors of the *first visit interval* (number of days between the first and second visits)^a^

Variable^b^	Est.	S.E.	*p*	Incidence Rate Ratio	95% Confidence Interval		Predicted First Interval
					Lower	Upper	
Intercept	2.70	0.08	<.0001	.	.	.	.
Age	−0.01	0.00	<.0001	0.99	0.99	0.99	Age 25 = 12.5
							Age 40 = 10.8
Receiving social assistance:							
Yes	0.07	0.03	0.01	1.07	1.01	1.14	12.5
No	(ref)	.	.	1.00	.	.	11.7
Alcohol problems:							
Yes	0.09	0.03	0.001	1.09	1.03	1.16	12.6
No	(ref)	.	.	1.00	.	.	11.6
Opioid problems ^c^:							
Yes	0.10	0.03	0.0005	1.11	1.04	1.17	12.7
No	(ref)	.	.	1.00	.	.	11.5
Frequency of substance use, past 30 days:							
Binge use	−0.13	0.07	0.07	0.87	0.77	1.01	11.4
3 times per week to daily	−0.13	0.03	<.0001	0.88	0.83	0.93	11.4
Up to 2 times per week	−0.03	0.04	0.41	0.97	0.90	1.05	12.6
No use	(ref)	.	.	1.00	.	.	13.0
Past-year injection drug use:							
Yes	−0.14	0.04	0.001	0.87	0.80	0.94	11.3
No	(ref)	.	.	1.00	.	.	13.0
Treatment mandate:							
Legal	0.25	0.06	<.0001	1.29	1.14	1.44	14.4
Child protection	0.07	0.03	0.02	1.07	1.07	1.01	12.0
Employer/school	−0.01	0.08	0.91	0.99	1.01	1.14	11.1
None	(ref)	.	.	1.00	.	.	11.2

^a^Negative binomial regression with random effect for agency; includes one episode per client (first episode selected). *N* = 4200. Excludes 662 clients (12.8% of 5162) whose first episode consisted of only 1 visit, and excludes 300 clients (5.8% of 5162) with > 1 visit but missing values on predictor variables. AIC = 29,021.6, BIC = 29,040.8

^b^Coefficients were not statistically significant for: education, employment status, marital status, cannabis problem, stimulant problem, legal system involvement, pregnant at admission

^c^Includes prescription opioids, heroin, opium, and over-the-counter codeine

Older clients were retained for longer: women aged 40 were predicted to stay in treatment for 11 days longer than were women aged 25 (Table 4). Relative to those who had not graduated high school, retention was predicted to be 11 days longer among women with a high school diploma. Problems with stimulants predicted an additional 26 days of retention. Women who were pregnant were predicted to stay in treatment 12 days longer than those who were not pregnant (or were unsure).

**Table 4:** Predictors of *treatment retention* (number of days between the first and last visit) ^a^

Variable^b^	Est.	S.E.	*p*	Incidence Rate Ratio	95% Confidence Interval		Predicted Retention Time
					Lower	Lower	
Intercept	4.44	0.16	<.0001	.	.	.	.
Age	0.01	0.00	0.04	1.00	1.01	1.01	Age 25 = 107.3
							Age 40 = 118.4
Education:							
Less than high school	−0.10	0.04	0.02	0.90	0.84	0.98	109.1
High school or more	(ref)	.	.	1.00	.	.	120.1
Stimulant problems^c^:							
Yes	0.23	0.04	<.0001	1.26	1.16	1.36	128.2
No	(ref)	.	.	1.00	.	.	102.2
Pregnant at admission:							
Yes	0.11	0.05	0.03	1.12	1.01	1.23	120.8
No, possibly, unknown	(ref)	.	.	1.00	.	.	108.4

^a^Negative binomial regression with random effect for agency; includes one episode per client (first episode selected). *N* = 5002. Excludes 160 clients (3.1% of 5162) with missing values on predictor variables. AIC = 55,673.0, BIC = 55,682.8

^b^Coefficients were not statistically significant for: receiving social assistance, employment status, marital status, alcohol problem, cannabis problem, opioid problem, frequency of substance use, legal system involvement, treatment mandate, pregnant at admission

^c^Includes powder cocaine, crack, other amphetamines including methamphetamine

Older age was also associated with higher treatment intensity: women aged 40 were predicted to have 3 more visits, on average, than were women aged 25 (Table 5). Women who did not have a high school diploma were predicted to have 2 fewer visits compared to women who had graduated high school. Receiving social assistance and being unmarried were associated with 1 additional visit (relative to the reference categories for these variables). Problems with stimulants predicted 2.7 additional visits. Past-month binge use and (near) daily substance use were associated with 3 and 1 more visits, respectively (relative to no past-month use). Women mandated by the legal system were predicted to have 2 fewer visits (relative to those who were not mandated). Women who were pregnant were predicted to have 2.5 additional visits (relative to those who were not pregnant or unsure if they were pregnant).

**Table 5:** Predictors of *treatment intensity* (number of visits per episode) ^a^

Variable^b^	Est.	S.E.	*p*	Incidence Rate Ratio	95% Confidence Interval		Predicted Number
					Lower	Upper	of Visits per Episode
Intercept	1.84	0.16	<.0001	.	.	.	.
Age	0.02	0.00	<.0001	1.02	1.02	1.02	Age 25 = 11.8
							Age 40 = 14.8
Education:							
Less than high school	−0.16	0.03	<.0001	0.85	0.80	0.90	11.8
High school or more	(ref)	.	.	1.00	.	.	13.8
Receiving social assistance:							
Yes	0.11	0.04	0.002	1.12	1.03	1.21	13.5
No	(ref)	.	.	1.00	.	.	12.1
Marital status:							
Not married	0.08	0.03	0.03	1.08	1.02	1.15	13.3
Married or partnered	(ref)	.	.	1.00	.	.	12.3
Stimulant problems ^c^:							
Yes	0.21	0.03	<.0001	1.23	1.16	1.31	14.2
No	(ref)	.	.	1.00	.	.	11.5
Maximum frequency of substance use, past 30 days:							
Binge use	0.24	0.10	0.01	1.27	1.04	1.55	15.0
3 times per week to daily	0.09	0.04	0.02	1.09	1.01	1.18	13.0
Up to 2 times per week	−0.03	0.05	0.60	0.98	0.88	1.07	11.6
No use	(ref)	.	.	1.00	.	.	11.8
Treatment mandate:							
Legal system	−0.19	0.08	0.02	0.83	0.71	0.97	10.7
Child protection services	0.04	0.04	0.25	1.04	0.96	1.13	13.5
Employer or school	0.10	0.10	0.34	1.11	0.91	1.34	14.3
None	(ref)	.	.	1.00	.	.	13.0
Pregnant at admission:							
Yes	0.19	0.04	<.0001	1.21	1.12	1.31	14.1
No, possibly, unknown	(ref)	.	.	1.00	.	.	11.6

^a^Negative binomial regression with random effect for agency; includes one episode per client (first episode selected). *N* = 4724. Excludes 438 clients (8.5% of 5162) with missing values on predictor variables. AIC = 33,837.2 BIC = 33,858.2

^b^Coefficients were not statistically significant for: employment status, alcohol problem, cannabis problem, opioid problem, legal system involvement, injection drug use

^c^Includes powder cocaine, crack, other amphetamines including methamphetamine

## Discussion

The goals of this study were to describe the population of women attending integrated programs in Ontario, and to evaluate levels and predictors of participation in treatment. Overall, this population of women was facing numerous barriers to accessing the resources and opportunities needed for health: in addition to being pregnant or new mothers experiencing problematic substance use, most were unemployed, on social assistance, and single. Despite this, programs appeared to be able to successfully engage most women, once they were admitted to treatment. Although rates of treatment participation did vary across subgroups defined by sociodemographic and admission characteristics, effect sizes tended to be small on average, providing little evidence in general of sociodemographic inequities in participation.

The high rates of participation among these integrated programs are encouraging. Although we were unable to identify the reasons underlying the high participation rates with these administrative data, findings from other parts of our evaluation of these programs provide further context. Coordination across agencies and sectors at the levels of service delivery and policy was seen to form a key part of what constitutes effective integrated care for this population [35], and may contribute to how these programs are able to maintain engagement among the women who access them. Qualitative investigation of women’s perspectives of these programs revealed the central role played by counsellor support for the emotion regulation and executive functioning features of the therapeutic relationship [38] – components that have been found elsewhere to link with positive outcomes [44, 45]. Factors such as multi-sectoral service coordination and therapeutic supports for emotion regulation and executive functioning may be particularly important for pregnant and parenting women who are accessing substance use services, given that they face numerous social and structural barriers to health (e.g., poverty, substance-related stigma, gender discrimination) [46–49].

The programs in our study were open-ended, rather than having a designated length, and we found that women continuously attended for an average of 125 days, or 4 months. This is comparable to lengths of stay for integrated programs reported elsewhere [1]. Further, we extend the literature by including counts of visits and the length of time between the first and second visit. In this suite of programs, women attended appointments about once a week for the 4 months that they were engaged. In the absence of data on client outcomes, it is difficult to speculate on the clinical significance of visit frequency for this population. There is some evidence from the mental health literature that weekly sessions of psychotherapy are associated with faster improvements in mental health (no data on specific diagnoses were available in this study) [50]. Weekly sessions have also been recommended in clinical guidelines for psychotherapeutic interventions for anxiety disorders [51, 52].

Studies have shown that a shorter interval between visits early on in a treatment episode is associated with a higher rates of engagement or retention (Acevedo et al., 2015b; Lee et al., 2012). In the present study, the rate of drop out after a single visit was 14%, which is considerably lower than the 23–50% reported elsewhere in standard treatments [53, 54]. Maintaining participation in treatment is a perennial challenge in substance use treatment generally [55, 56], and high rates of drop-out in the initial weeks of treatment is one signal that programs are not meeting the needs and/or expectations of clients. To the extent that longer retention is associated with positive outcomes in the longer term (e.g., reduced substance use, arrests, and incarceration) [15–17, 57], then such measures offer up important information on program performance. Our findings suggest that these integrated programs have achieved a fair degree of success in at least engaging women in services after admission. Further work is planned evaluating the link between these indicators of engagement and maternal and child health outcomes.

Examining the predictors of participation provides insight into variation across the treatment population, and whether additional efforts are required to meet the needs of specific subgroups. Adjusting for substance use and other admission characteristics, older age consistently predicted better participation. Women also stayed in the program longer and attended more visits if they had a high school diploma or were pregnant. These findings differ from what has been reported previously in that high income, being married and unemployed were associated with longer retention in women [31]. In the present study, the magnitude of the association between these characteristics and participation outcomes was small (i.e., incident rate ratio < 2.0) [58]. Nonetheless, as systems and services continue to evolve to support women, results suggested that younger women and women with lower education may need additional supports for participation. Further, pregnancy at admission predicted longer retention and greater intensity, yet only a small minority of women was pregnant when they entered treatment. Given the potential for better maternal and child outcomes associated with earlier engagement in integrated programs (including prenatal care and support for the social determinants of health, as well as substance use treatment), additional outreach efforts may be warranted to engage women while they are pregnant [11, 46]. There is a need for qualitative studies of the ways in which barriers to care are experienced across the population, attending to the intersections between identities, and how these impact on participation on integrated treatment (e.g., [59, 60]). Further, systematic quantitative exploration and statistical modeling of the impacts of structural violence at multiple levels (individual, community, and population) is also needed to inform the development of policies and practices that both promote health and reduce health disparities.

Our findings join a growing body of research in supporting the use of integrated (comprehensive and holistic) service models for women who have problematic substance use, and suggests that efforts to scale up women-focused programs are warranted [1, 7, 9–13]. As noted earlier, with only 36 such programs operating across the province, most Ontario communities do not offer integrated substance use services for pregnant and parenting women. National studies in Canada have identified gaps in the capacity of substance use treatment agencies to offer comprehensive services addressing maternal and child health [61]. With a narrowing gender gap in rates of substance use and related problems in many countries [62–66], effective programming for women has myriad public health implications for women’s health and well-being, as well as fetal and child development. There is a need for dedicated attention and resources to support the evolution of substance use service systems as they work to ensure that they are able to address the unique contexts of substance use among women.

In this study, mandates from the legal system or child protection services tended to be associated with lower participation. Specifically, both types of mandates were associated with a prolonged interval between the first and second visits, and legal mandates were associated with a lower overall number of visits. Previous findings on the association between mandates and retention are equivocal: some studies have reported that legal and employer mandates are associated with prolonged retention [26, 67, 68], while others have found that mandates are associated with higher dropout rates [69, 70]. Research into the effectiveness of mandated treatment has emphasized mandates from the legal system; however, only 4.3% of the women in this treatment population were mandated to treatment by the legal system. There is limited work examining mandates from child protection services, and their impact on treatment processes and outcomes. Given the key role that such mandates play in promoting treatment entry in this population (i.e., over one in four women in this population was mandated through child protection services), these issues deserve further attention.

Strengths of this study are its population focus and the inclusion of a suite of integrated programs embedded within a broader system of psychosocial treatment for substance use. That said, the data source excluded private treatment and substance use services received outside of the publicly funded system of specialized psychosocial services. The measures of sociodemographic characteristics and substance use were self-reported and are, therefore, subject to potential reporting and recall biases. As with any secondary analysis of administrative data, not all potentially important predictor variables were available; specifically, we lacked measures of rural versus urban location, mental health problems, previous treatment experiences, race and ethnicity, and poly-substance use (over and above the use of substances self-reported to be causing problems). For the subset who were pregnant at admission, we have no information on how far along the women are in their pregnancies. There was also no information on family structure or custody. Previous work reported that having more than two children predicted earlier drop out from treatment [71]. Finally, because this study is based on data from a treatment system, we are unable to address issues of access to integrated programs. Although we found only weak associations between sociodemographic variables and participation once women had entered these programs, it is nonetheless possible that inequities exist across population subgroups in access to services in the first place.

## Conclusions

This study is the first system-level study to describe the clientele and rates and predictors of participation in integrated programs for pregnant and parenting women. Services appeared to have achieved reasonable levels of success in engaging women in treatment once they were in the program, and there was little evidence of sociodemographic inequities in participation. Future directions for research include studying the influence of program-level factors (e.g., provision of child care, case management, prenatal care, case coordination with child protection services) on participation, and on how these relate to maternal and child outcomes.

## Abbreviations

AIC: Akaike information criterion
BIC: Bayesian information criterion
DATIS: Drug and Alcohol Treatment Information System
